# Characterization of the monoclonal antibody and the immunodominant B-cell epitope of African swine fever virus pA104R by using mouse model

**DOI:** 10.1128/spectrum.01401-23

**Published:** 2024-02-02

**Authors:** Qichao Chen, Lixinjie Liu, Shibang Guo, Liang Li, Yifeng Yu, Zhankui Liu, Chen Tan, Huanchun Chen, Xiangru Wang

**Affiliations:** 1 National Key Laboratory of Agricultural Microbiology, College of Veterinary Medicine, Huazhong Agricultural University, Wuhan, China; 2 Key Laboratory of Preventive Veterinary Medicine in Hubei Province, The Cooperative Innovation Center for Sustainable Pig Production, Wuhan, China; 3 Key Laboratory of Prevention & Control for African Swine Fever and Other Major Pig Diseases, Ministry of Agriculture and Rural Affairs, Wuhan, China; 4 International Research Center for Animal Disease, Ministry of Science and Technology of the People’s Republic of China, Wuhan, China; Iowa State University College of Veterinary Medicine, Ames, lowa, USA

**Keywords:** African swine fever virus, pA104R, monoclonal antibody, immunodominant B-cell epitope

## Abstract

The African swine fever virus (ASFV) structural protein pA104R is the only histone-like protein encoded by eukaryotic viruses. pA104R is an essential DNA-binding protein required for DNA replication and genome packaging of ASFV, which are vital for pathogen survival and proliferation. pA104R is an important target molecule for diagnosing, treating, and immune prevention of ASFV. This study characterized monoclonal antibodies (mAbs) against pA104R and found them to recognize natural pA104R in ASFV strains with different genotypes, showing high conservation. Confirmation analyses of pA104R epitopes using mAbs indicated the presence of immunodominant B-cell epitopes, and further characterization showed the high antigenic index and surface accessibility coefficients of the identified epitope. Furthermore, the pA104R protein functions through the polar interactions between the binding amino acid sites; however, these interactions may be blocked by the recognition of generated mAbs. Characterizing the immunodominant B-cell epitope of the ASFV critical proteins, such as pA104R, may contribute to developing sensitive diagnostic tools and vaccine candidate targets.

**IMPORTANCE:**

African swine fever (ASF) is a highly pathogenic, lethal, and contagious viral disease affecting domestic pigs and wild boars. As no effective vaccine or other treatments have been developed, the control of African swine fever virus (ASFV) relies heavily on virus detection and diagnosis. A potential serological target is the structural protein pA104R. However, the molecular basis of pA104R antigenicity remains unclear, and a specific monoclonal antibody (mAb) against this protein is still unavailable. In this study, mAbs against pA104R were characterized and found to recognize natural pA104R in ASFV strains with different genotypes. In addition, confirmation analyses of pA104R epitopes using mAbs indicated the presence of immunodominant B-cell epitopes, and further characterization showed the high antigenic index and surface accessibility coefficients of the identified epitope. Characteristics of the immunodominant B-cell epitope of ASFV proteins, such as pA104R, may contribute to developing sensitive diagnostic tools and identifying vaccine candidate targets.

## INTRODUCTION

African swine fever (ASF), caused by the African swine fever virus (ASFV), is a highly pathogenic, lethal, and contagious viral disease affecting domestic pigs and wild boars of any breed and age (1, 2). Clinically, ASF mainly manifests as acute hyperthermia and causes bleeding in the reticuloendothelial system and is a contact-spreading disease with high morbidity and mortality (3). ASFV infection in wild boars was first reported in Kenya in 1921 and later spread to other regions (4, 5). After widespread circulation, ASFV has spread rapidly to countries worldwide, including Europe (6, 7) and Asia (8, 9), and has caused a substantial economic impact on the pig industry and threatened global food security (10, 11).

ASFV is a giant, enveloped, double-stranded DNA virus (12) that possesses five layers of coating structure surrounding an icosahedral viral capsid (13, 14). Its genome varies from 170 to 190 kbp and encodes over 150 proteins (12, 15). These proteins are involved in viral infection, replication, immune escape, and host metabolic regulation (12, 16). Due to the large and complex structure of ASFV, there is a limited functional understanding of these viral proteins. Although approximately 70 structural proteins have been identified in viral particles, the function of most proteins is unknown and needs to be explored (17).

As no effective vaccines or other treatments have been developed or approved, the control of ASFV relies heavily on early virus detection and diagnosis of the virus-infected herds (18). The structural proteins that combine P72, P54, and P30 encoded by ASFV are the primary targets for serological diagnosis because of their high immunogenicity and conservation, which forms the basis of current commercial diagnosis (19–21). Another potential serological target is the structural protein of 104 amino acids, pA104R, which is the product of the viral gene A104R, located in the nucleoid and is related to nucleoid assembly (13). pA104R is one of the main structural proteins of ASFV and has been shown to function as a histone-like protein closely associated with viral DNA in the virus particle (22). As the only histone-like protein encoded by eukaryotic viruses, pA104R is essential for viral DNA replication, transcription, and viral genome packaging (23). In addition, the pA104R sequence contains a histone-like protein signature and plays an essential role in promoting stable, organized, compact nucleoid and heterochromatinization of the host cell genome (22, 24). As mentioned earlier, heterochromatinization may lead to the silencing of particular host genes as part of an effective counteracting host immune response, contributing to viral infection (25).

Previous studies have shown that pA104R possesses strong antigenicity and immunogenicity and is a promising target for immunoglobulin IgM/G antibody responses (26). Generally, swine infected with ASFV develop antiviral antibodies from 7 to 10 days post-infection, persisting for extended periods (27). A consistently high antibody response against the histone-like protein pA104R has been observed in infected animals (28). Notably, some researchers have established an enzyme-linked immunosorbent assay (ELISA) detection method. A pA104R-specific ELISA was employed to detect the serum of ASFV antibodies, and the results were substantially aligned with the results of the “gold standard” immunoblotting test (29). These properties make pA104R an ideal antigen for serological diagnostic tools to evaluate ASFV infection and its incorporation into vaccines. However, the molecular basis of pA104R antigenicity remains unclear, and a specific monoclonal antibody (mAb) against this protein is still unavailable, limiting the application and basic research on pA104R.

It is well known that hosts generate adaptive immune responses by recognizing antigenic epitopes to resist foreign pathogens' invasion. Therefore, a detailed epitope analysis is essential for understanding immunological events and immune protection mechanisms and developing epitope-based diagnostic tools and vaccines for various diseases (30, 31). In this study, the recombinant protein pA104R was applied as an immunogen to generate mAbs through the hybridoma technique. Seven mAbs were prepared and characterized, and two of these mAbs with potential diagnostic applications could recognize well with the ASFV strain. Moreover, we identified the novel immunodominant B-cell epitopes of pA104R and detected strong antibody responses to these epitopes in swine infected with ASFV. Overall, our findings provide a basis for further investigation of pA104R in the immune response during the infection, which shall improve the ASFV diagnosis and develop effective vaccines against this disease.

## RESULTS

### ASFV pA104R recombinant protein preparation and immunization

**Fig 1:**
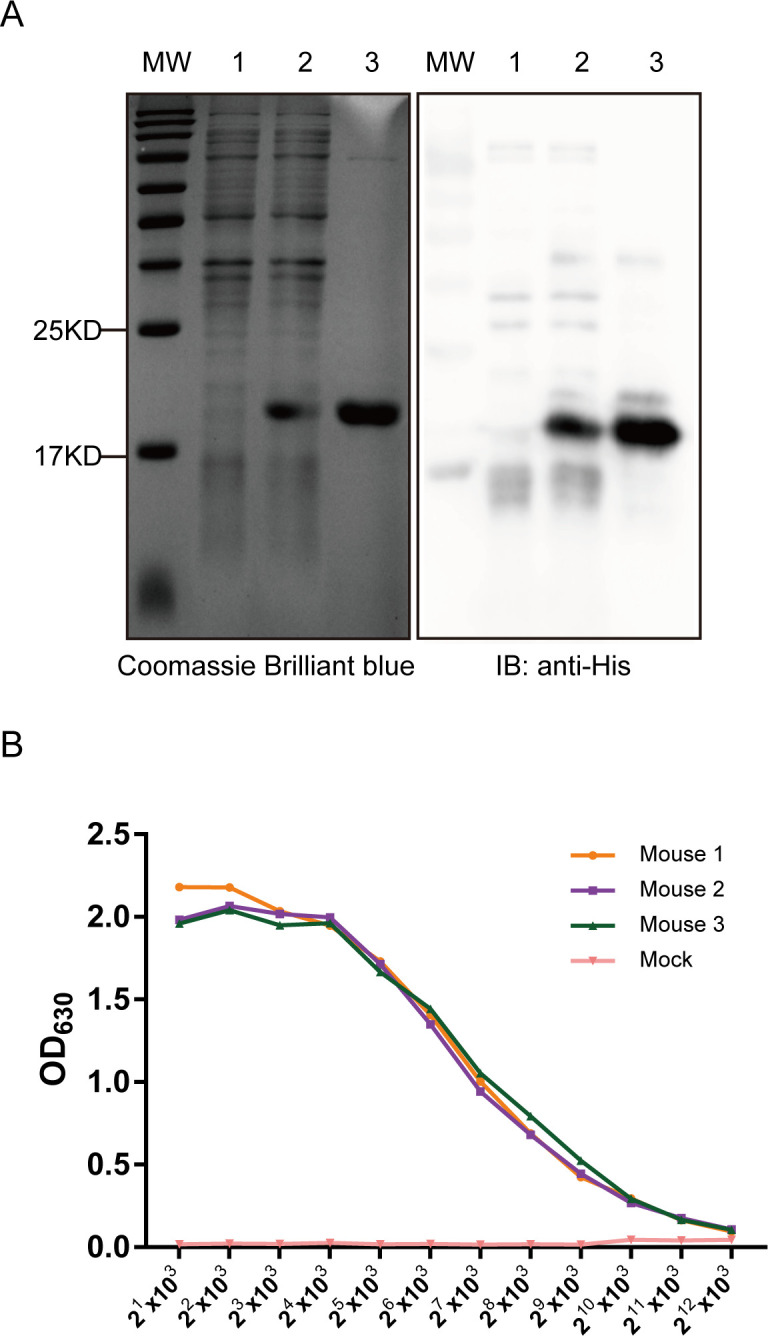
Preparation and immunization of pA104R recombinant protein. The purified recombinant pA104R protein was subjected to SDS-PAGE separation. Subsequently, the gel was stained with Coomassie Brilliant blue to visualize the protein bands. Immunoblotting was performed using an anti-His antibody. In the image, lane 1 represents the pET-30a vector with IPTG, lane 2 represents the pET-30a-A104R with IPTG induction, and lane 3 represents the purified His-pA104R protein (**A**). The molecular weight (MW) of the protein was determined. Mouse serum titers against the pA104R protein were evaluated using an indirect ELISA after three immunization rounds (**B**).

The pA104R sequence was amplified by PCR and ligated into a prokaryotic expression vector to generate the recombinant plasmid pET-30a-A104R. After induction, the recombinant His-fused pA104R protein was successfully expressed in *Escherichia coli* BL21(DE3) cells. The recombinant pA104R protein was purified by Ni-NTA agarose, verified by polyacrylamide gel electrophoresis (SDS-PAGE), and immunoblotting with a His-tagged antibody (Fig. 1A). Subsequently, female BALB/c mice were subjected to three immunizations using the purified pA104R recombinant protein, with a 2-week interval between each immunization. The mice were subjected to euthanasia for subsequent cell fusion and mAb preparation once the titers of antibodies against pA104R reached sufficiently high levels (Fig. 1B).

### Identification and characterization of the anti-pA104R mAb

**Fig 2:**
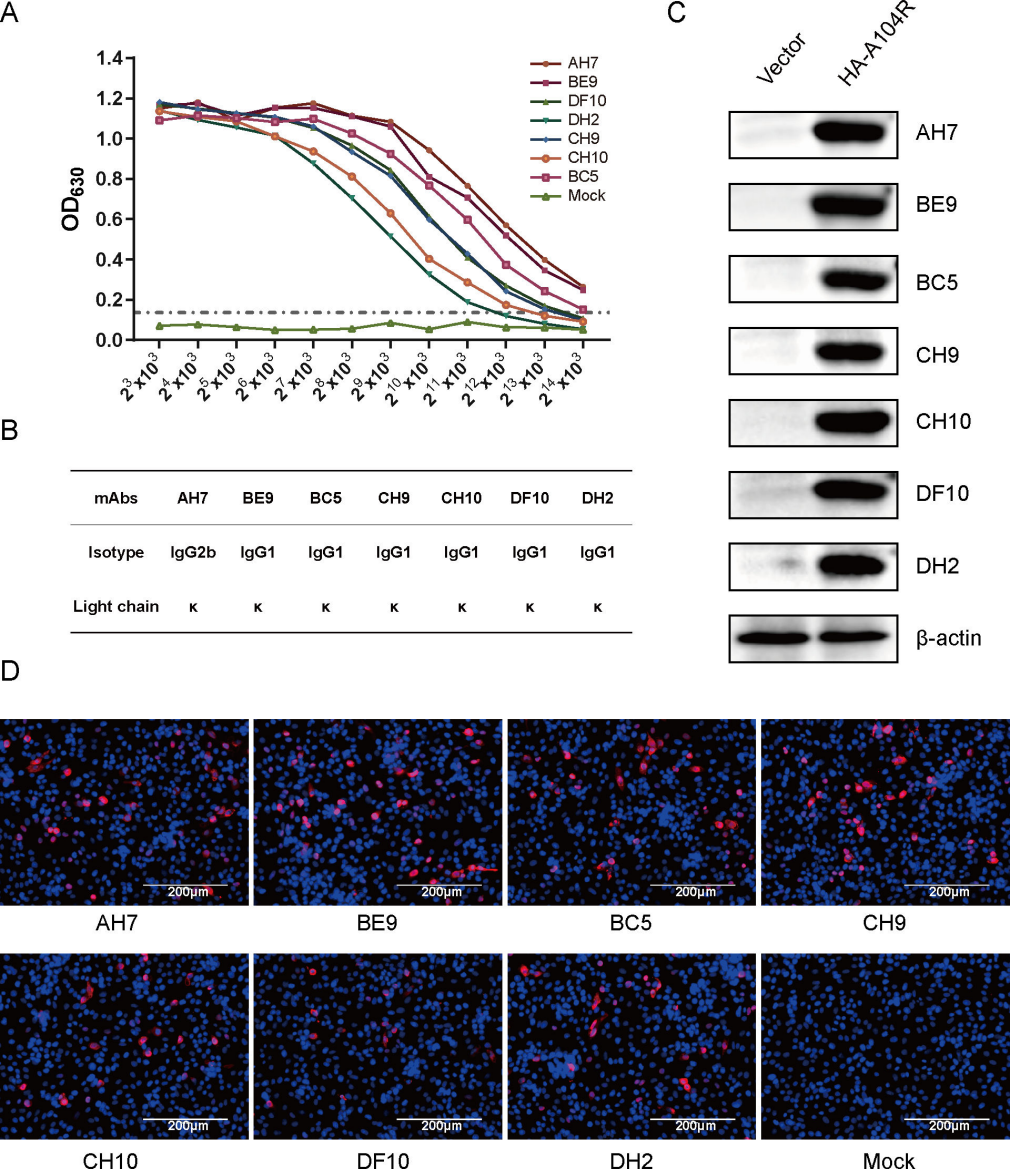
Characterization of mAbs against pA104R. The seven mAbs were produced using the ascites method, and the antibody titer of ascites fluid was determined by indirect ELISA (**A**). Isotype identification of the seven mAbs was performed (**B**). For further characterization, the reactivity of the seven mAbs was assessed using eukaryotic-expressed pA104R protein obtained by transfecting HEK-293T (**C**) and PK-15 (**D**). Immunoblotting was conducted to examine the binding of the mAbs to the pA104R protein (**C**). An indirect immunofluorescence assay was carried out using these mAbs, with the resulting fluorescence indicating the binding of the antibodies to the pA104R protein (**D**). The scale bar in the immunofluorescence assay image represents 200 µm.

After being subcloned 3–5 times by limiting dilution and testing of hybridoma cell supernatants with indirect ELISA, seven stably positive clones were finally identified and designated as AH7, BE9, BC5, CH9, CH10, DF10, and DH2, respectively. These positive clones were subsequently used to prepare ascites containing mAbs, all of which yielded high antibody titers, especially for AH7 and BE9 (Fig. 2A). Next, the subtype of each mAb was identified. All mAbs belonged to the IgG class combined with kappa light chains (κ), with AH7 belonging to the IgG2b subclass and the remaining antibodies belonging to the IgG1 subclass (Fig. 2B). The specificity of these mAbs was confirmed by immunoblotting and indirect immunofluorescence assay (IFA) with the eukaryotic expression plasmid pCAGGS-HA-pA104R transfected into HEK-293T cells (Fig. 2C) or PK-15 cells (Fig. 2D). As shown, all seven mAbs reacted explicitly with the eukaryotic-expressed pA104R protein.

### Reactivity of anti-pA104R mAbs with native ASFV protein

**Fig 3:**
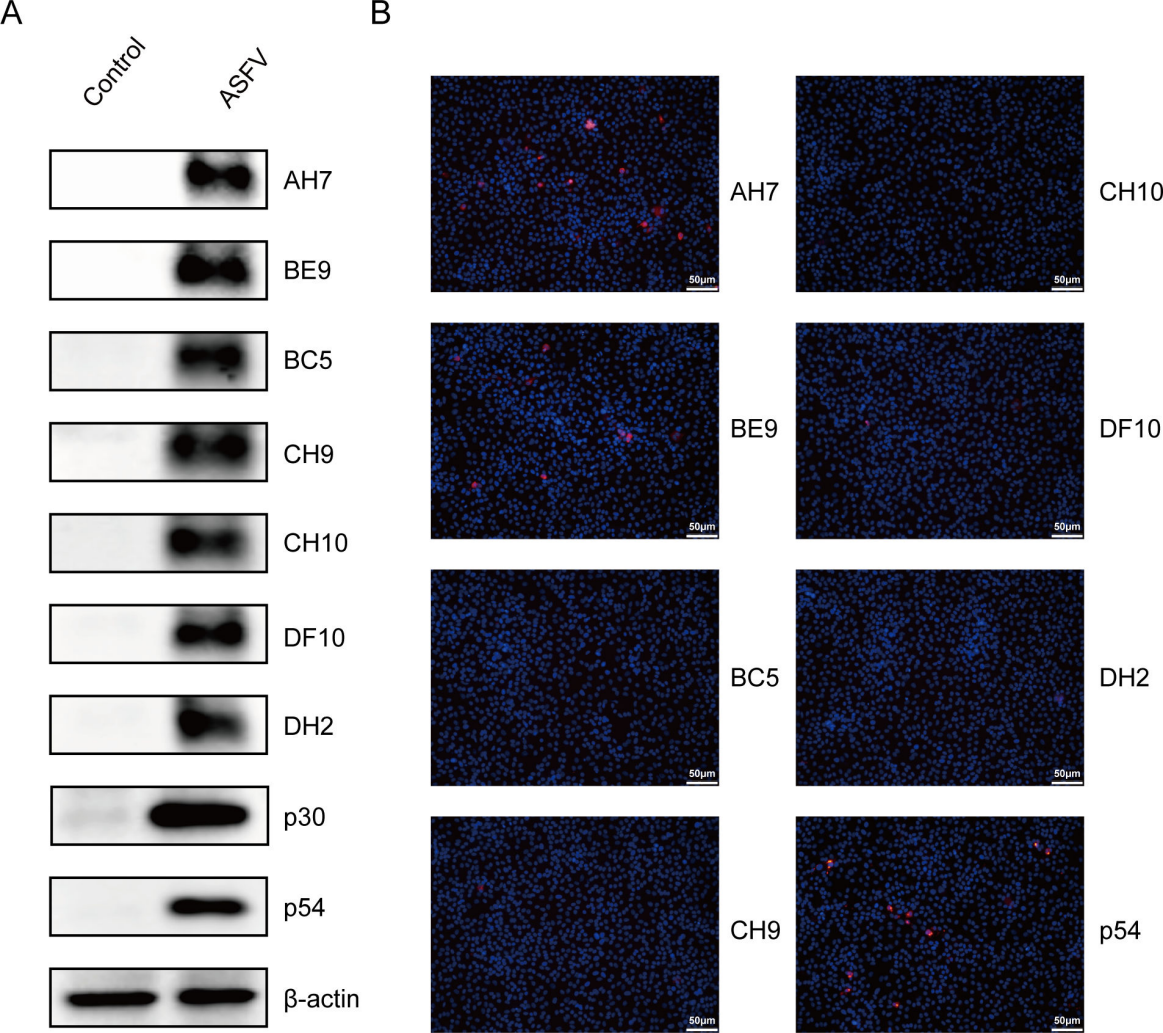
Reactivity of mAbs with native ASFV pA104R protein. To assess the reactivity of the mAbs with the native ASFV pA104R protein, porcine alveolar macrophage and wild boar lung cells were infected with ASFV at 0.1 MOI for 48 h. Immunoblotting (**A**) and an indirect immunofluorescence assay (**B**) were performed to examine the binding of the mAbs to the native pA104R protein. The scale bar in the immunofluorescence assay image represents 200 µm and 50 µm.

To further determine the natural pA104R antigenic specificity, the mAbs were subjected to immunoblotting and IFA in the infected samples. The ASFV-infected cells were analyzed using those seven mAbs as primary antibodies to determine their antigenic reactivity and specificity. As demonstrated, the immunoblotting results showed that all seven mAbs could react with the denatured ASFV pA104R protein in infected cells (Fig. 3A), indicating all mAbs could recognize the linear epitopes of pA104R. The antibodies against p30 and p54 were used to confirm ASFV infection. However, unexpectedly, the IFA showed different results for these seven mAbs. Only mAbs AH7 and BE9 could bind to the undenatured ASFV pA104R antigen in the cells (Fig. 3B), while the others could not, suggesting that mAbs AH7 or BE9 could specifically recognize the spatial epitope of native ASFV pA104R protein in the infected samples.

### Determination of pA104R immunodominant epitope with mAbs

**Fig 4:**
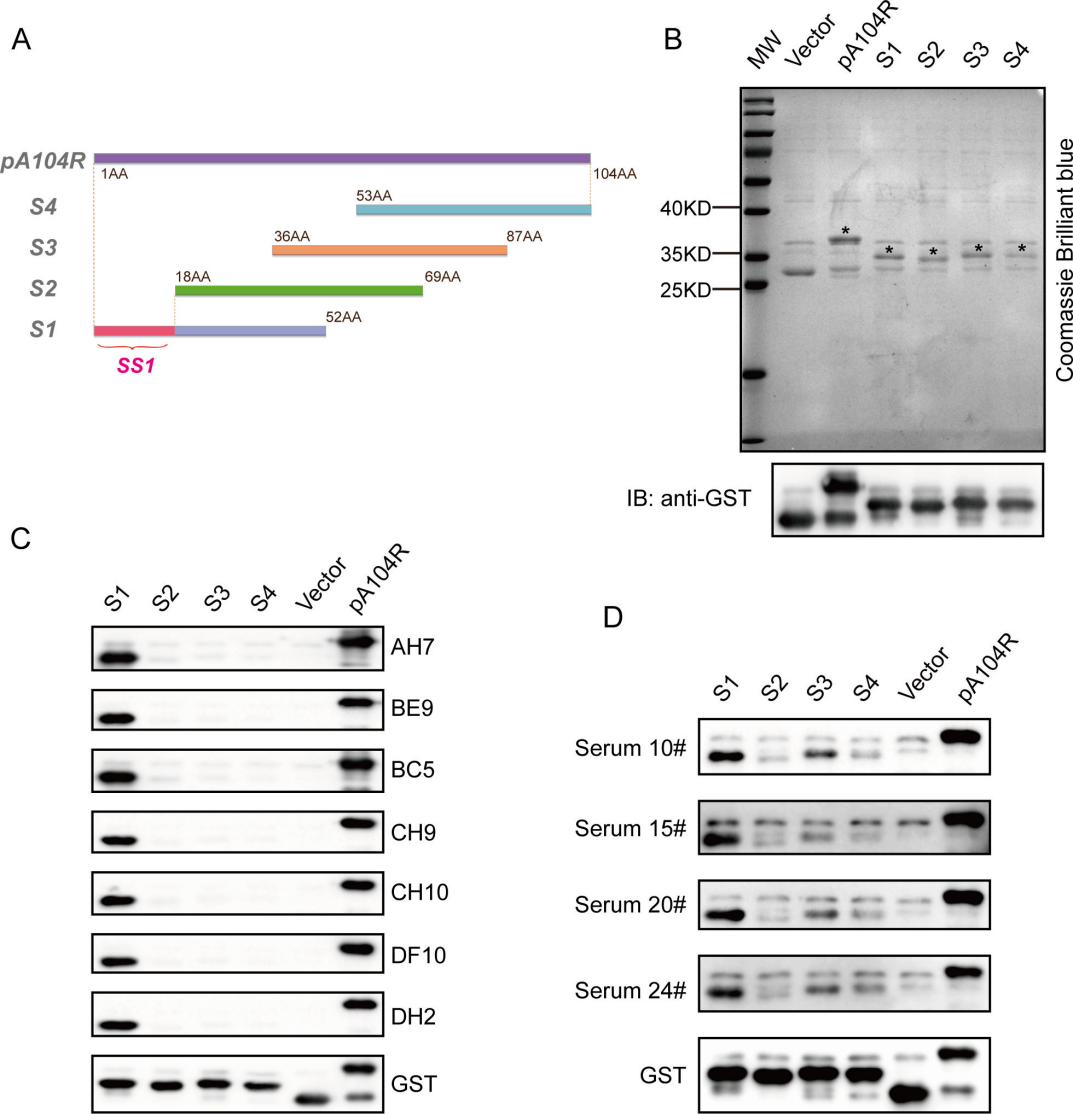
Identification of antigenic epitopes of ASFV pA104R. A schematic diagram was created to illustrate the locations of these truncated fragments (**A**). Each truncated recombinant protein was then separated using SDS-PAGE and stained with Coomassie Brilliant blue to visualize the protein bands (**B**). Immunoblotting was performed using an anti-GST antibody to confirm the identity of the truncated fragments (**B**). Immunoblotting was also conducted to assess the reactivity of the truncated fragments (S1–S4) with the anti-pA104R mAbs (**C**). The immunodominant epitope of pA104R was further validated by immunoblotting with different ASFV-positive serums (**D**).

To identify the antigenic epitope recognized by each anti-pA104R mAb, four truncated and overlapping recombinant proteins (S1–S4) spanning the pA104R protein were designed and prokaryotic-expressed, schematically shown in Fig. 4A. The expressed pA104R truncated recombinant proteins (S1–S4) were verified by SDS-PAGE and immunoblotting with a GST (Glutathione S-transferase)-tagged antibody (Fig. 4B). Next, immunoblotting was used to determine the immune reactivity between the seven mAbs with pA104R truncated proteins. Interestingly, all mAbs recognized only the full-length pA104R protein or the truncated protein S1 rather than S2, S3, and S4 (Fig. 4C), implying that all mAbs identified a region located in the unique area of S1 herein we called SS1 (Fig. 4A). We, therefore, hypothesized that SS1 region might be an immunodominant epitope of the pA104R protein. Four ASFV-positive serums were used to analyze the reaction with the pA104R truncated proteins to verify this concept. The immunoblotting results supported that all clinically positive serums could react strongly with the pA104R protein. More importantly, the S1 region was the primary antigenic epitope recognized by the antibody (Fig. 4D), which further evidenced that SS1 is an immunodominant epitope of the ASFV pA104R protein.

### Preliminary application of the mAbs

**Fig 5:**
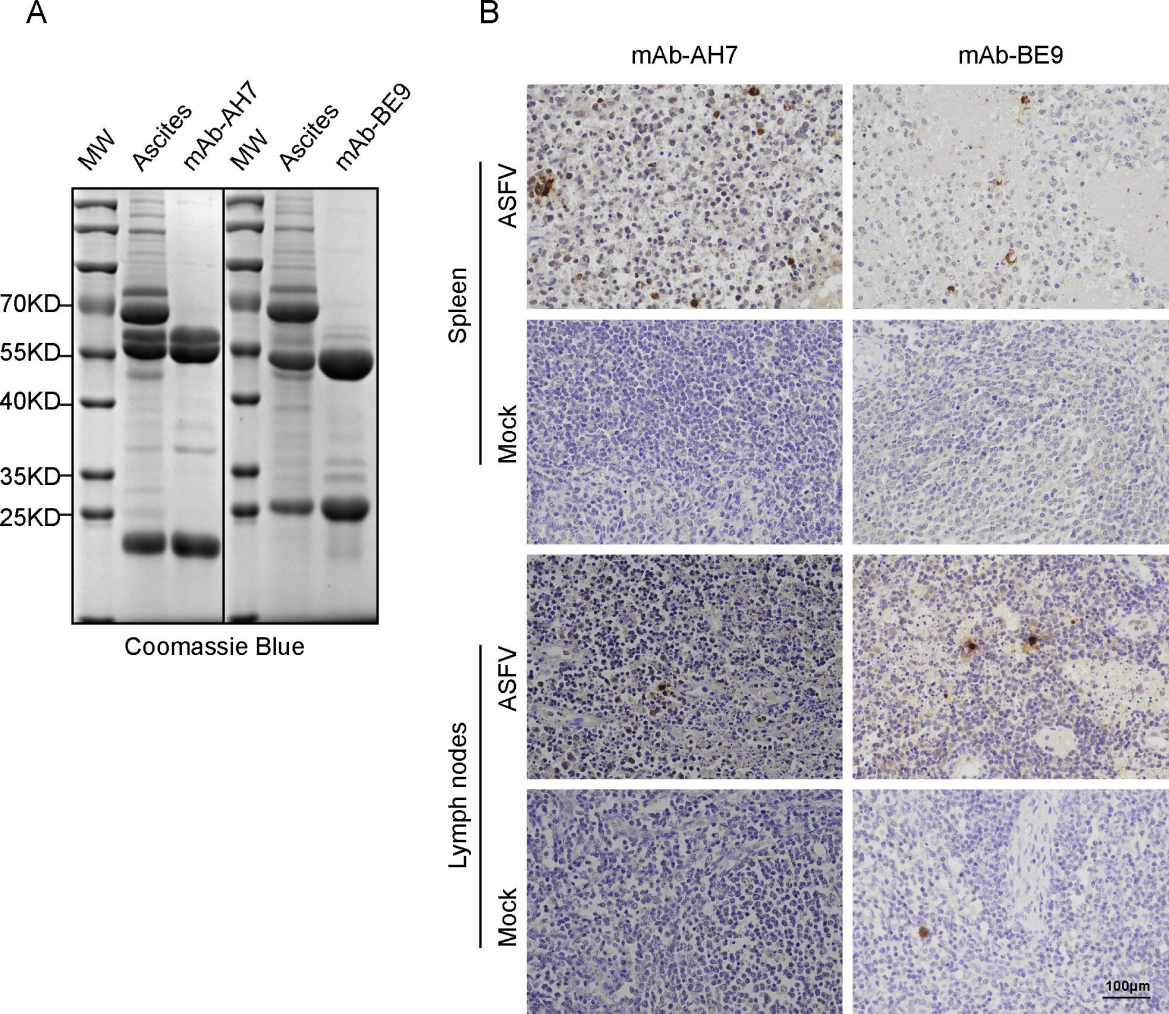
Immunohistochemical analysis of pA104R in ASFV-infected tissue samples by mAbs AH7 and BE9. The purified mAbs AH7 and BE9 were obtained from ascites fluid, and their purity was confirmed using SDS-PAGE stained with Coomassie Brilliant blue (**A**). Immunohistochemistry was performed on the sections of paraffin-embedded spleen and lymph nodes from ASFV-infected animals. The mAbs AH7 and BE9 were used as the primary antibodies to specifically bind to the pA104R within the ASFV-infected tissues (**B**). The scale bar in the images represents 100 µm.

Although all seven mAbs could identify ASFV-infected cells by immunoblotting, only AH7 and BE9 of the seven mAbs could be applied in the IFA assay (Fig. 3). Therefore, to investigate the potential applications of these mAbs, AH7 and BE9 were also subjected to immunohistochemical (IHC) assays to identify pA104R in ASFV-infected tissues. For better IHC performance, the AH7 and BE9 ascites were purified by protein G agarose and detected by SDS-PAGE analysis (Fig. 5A). The IHC analysis showed strong pA104R-specific signals in paraffin-embedded pig tissues incubated with AH7, whereas BE9 incubation showed relatively weak signs in the same tissues from ASFV-infected tissues. In contrast, no signaling was observed in mock tissues incubated with both mAbs (Fig. 5B).

### Spatial distribution of the pA104R immunodominant epitope

**Fig 6:**
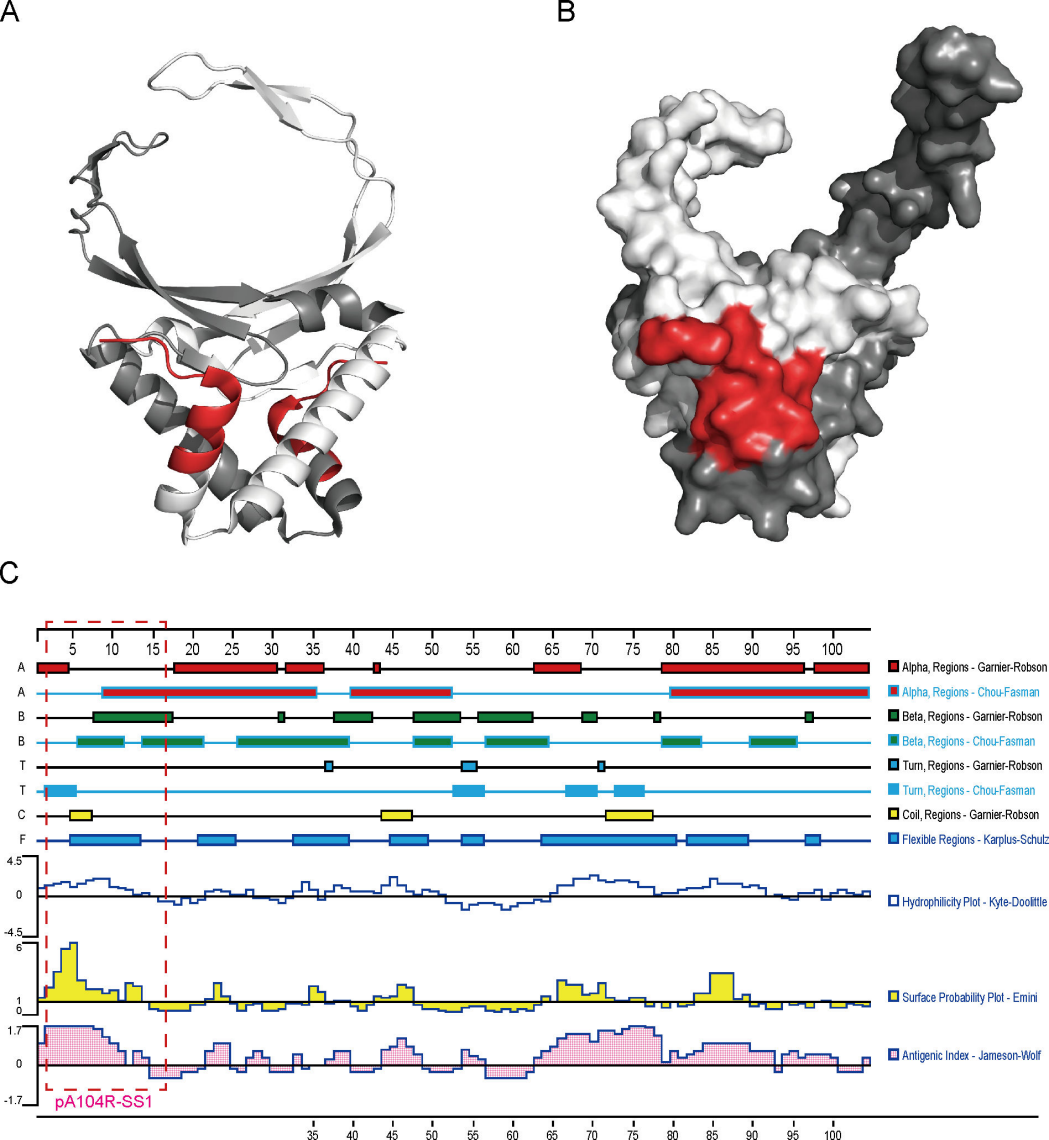
Bioinformatic characterization of the identified epitope in ASFV pA104R protein. The spatial localization of the SS1 epitope was determined using PyMol software, which generated a 3D structure model of the pA104R. The SS1 epitope is depicted in red, highlighting its position within the protein structure (**A and B**). Additionally, PROTEAN software was utilized to predict the second structural feature of the pA104R protein. The results were presented as a diagram, with the SS1 epitope enclosed within a red dashed-line box (**C**).

A 3D structural model of ASFV pA104R with ID 6LMH from the Protein Data Bank was obtained, and the immunodominant epitope SS1 was characterized by using bioinformatics. As shown, the epitope SS1 forms an α-helix in the secondary structure of the pA104R protein (Fig. 6A). Visualization of the spatial structure distribution revealed that the immunodominant epitope SS1 is exposed on the surface of the pA104R in the 3D structure model using PyMOL software (Fig. 6B). Moreover, by PROTEAN software analysis, the amino acid sequences of SS1 epitope showed high hydrophilicity, antigenic index, and surface accessibility coefficients (Fig. 6C), which is consistent with the results of the spatial structural analysis.

### Conservation of immunodominant epitope among different ASFV genotypes

**Fig 7:**
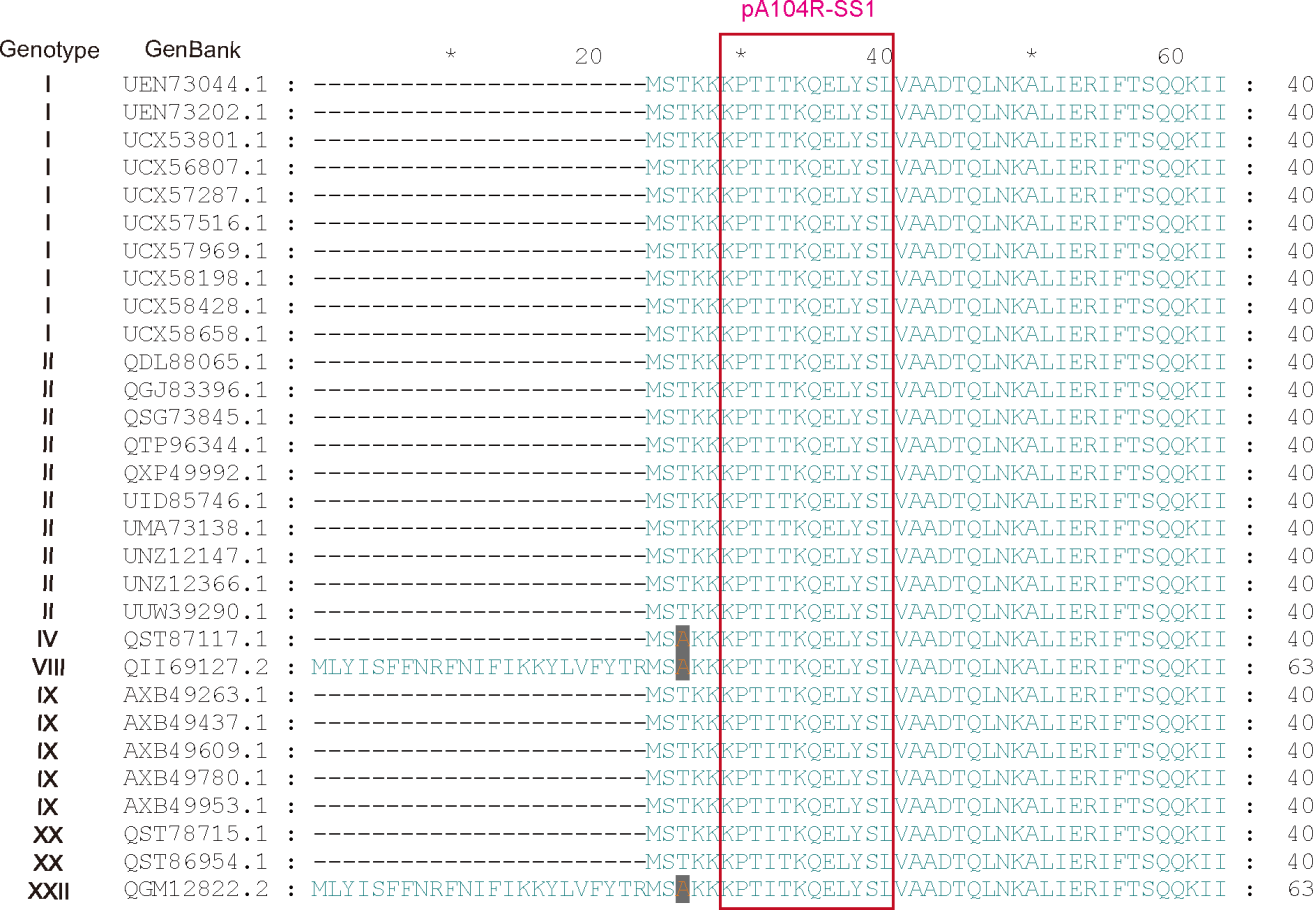
Evaluation of sequence conservation. The SS1 epitope of pA104R among different ASFV genotypes (genotypes I, II, IV, VIII, IX, XX, and XXII) was evaluated utilizing ClustalW for alignment and GeneDoc software for generating a visual depiction of the conservation level.

The immunodominant epitope of pA104R from 30 ASVF isolates with different genotypes (genotypes I, II, IV, VIII, IX, XX, and XXII) was compared using ClustalW in GeneDoc software to evaluate their epitope conservation among different ASFV genotypes. The amino acid alignment showed that epitope SS1 (marked in red box) was highly conserved among diverse ASFV strains with different genotypes (Fig. 7). These findings suggest that the mAbs AH7 and BE9, which specifically target the SS1 epitope in pA104R, have the potential to recognize all genotypes of ASFV strains. It implies that AH7 and BE9 mAbs could be developed as highly effective diagnostic reagents for detecting ASFV in clinical samples.

## DISCUSSION

In recent years, ASFV has spread rapidly and seriously and threatens global agricultural economic development. Previous studies have shown that humoral immunity plays an essential role in the protective immune response of pigs against ASFV (26). Regrettably, more research is needed on the exact mechanisms and range of critical serological viral immuno-determinants. According to previous functional analyses of ASFV, pA104R is essential for viral genome replication and packaging (23). Moreover, pA104R is a promising target for eliciting immunoglobulin responses due to its strong immunogenicity (26). However, the characterization of pA104R immunogenicity remains to be seen regarding how pA104R induces a humoral immune response in ASFV-infected pigs, and no B-cell epitope research on pA104R has been reported. Therefore, further understanding of the structure and antigenic properties of pA104R is urgently required for developing effective vaccines and immunodiagnostic approaches.

Within the scope of this research, our focus centered on the preparation of pA104R recombinant protein derived from genotype II ASFV. It is worth noting that this particular genotype holds significance as the prevailing endemic strain in China. Subsequently, we proceeded to elucidate the production of seven mAbs, namely AH7, BE9, BC5, CH9, CH10, DF10, and DH2, through the utilization of the purified pA104R as an immunogen (Fig. 1). IgG1 is the predominant subclass in mouse antiserum associated with T cell-dependent reactivity in response to Th2 cytokines. The T cell-independent response leads to the production of IgG2b antibodies (32, 33). These different mAbs subclasses (Fig. 2B) suggest that the epitope SS1 might act as both a thymus-dependent and independent antigen, which may trigger both humoral immunity and cellular immunity (34). The reactivity of the seven mAbs with eukaryotic-expressed pA104R proteins was confirmed by immunoblotting and IFA (Fig. 2C and D). Interestingly, the discrepancy in the reactivity of seven mAbs to ASFV-infected samples was observed in the IFA assay but did not appear in immunoblotting (Fig. 3), implying that ectopic prokaryotic expression might cause aberrant conformation or modification compared with the natural antigen. Significantly, the findings from this study provide evidence that mAbs AH7 and BE9 possess the ability to recognize the native conformation of the pA104R protein. This observation highlights their substantial potential for practical applications. It is important to note that epitopes play a crucial role in determining the antigenicity of viral proteins and in eliciting humoral immune responses (35). Interestingly, identifying mAb recognition epitopes showed that all seven mAbs reacted with the same antigenic region S1 (Fig. 4A and C), implying that this SS1 epitope is a B-cell immunodominant epitope of ASFV pA104R protein. The reaction extent of ASFV-positive serum with pA104R truncated protein also confirmed this speculation (Fig. 4B). The identification of epitopes within ASFV proteins contributes to a deeper comprehension of viral-host interactions and holds paramount importance in the development of efficacious vaccines and diagnostic tools.

Antibodies targeting immunodominant epitopes have been documented to facilitate viral clearance in infected hosts and play a crucial role in establishing protective immunity (36). This finding underscores the potential application of the SS1 epitope in developing ASFV vaccines. It is believed that the spatial localization of SS1 on the surface of the pA104R contributes to enhancing the immune response during ASFV infection (Fig. 6). Previous reports have indicated that E79 on the arm of pA104R forms polar contacts with K11 and Q12 of neighboring pA104R molecules. These opposing forces stabilize the interactions between multiple pA104R molecules, resulting in a durable continuous scaffold that can effectively hold the DNA (37). However, the amino acid sites K11 and Q12 of pA104R are coincidentally recognized and covered by anti-pA104R mAbs (Fig. 4). This covering may disrupt the polar interactions between multiple pA104R proteins and affect the normal function of pA104R, thereby inhibiting viral proliferation. In addition, the analysis demonstrated that mAb AH7 exhibited excellent performance characterized by high specificity and efficiency (Fig. 5B). This achievement enables the visualization of the *in vivo* distribution and localization of ASFV (38, 39). Additionally, it is noteworthy that this B-cell immunodominant epitope remains conserved across various ASFV genotypes (Fig. 7), further underscoring its immense potential for applications in vaccination and diagnosis.

In addition to the structural proteins P72, P54, and P30, the pA104R protein is considered a promising serologic target. It exhibits notable conservation and immunogenicity. Notably, infected animals consistently exhibit a robust antibody response against the histone-like protein pA104R (28). Furthermore, elevated levels of pA104R antibodies have been observed in asymptomatic animals compared to chronically infected pigs (26), suggesting a potential direct involvement of pA104R in immunoprotection. These characteristics position pA104R as an ideal antigen for serological diagnostic tools to assess ASFV infection and its integration into vaccine formulations.

In our previous investigation, we identified that pA104R possesses the ability to counteract the host’s innate immune response (40), thereby impacting its effectiveness as an immunological vaccine. Characterizing the immunodominant epitope of pA104R offers a means to overcome its inhibitory functional sites, enabling the full exploitation of its immunoprotective potential. In conclusion, we successfully generated mAbs targeting the pA104R of ASFV and elucidated its immunodominant B-cell epitope. These findings significantly contribute to our understanding of the intricate relationship between the structure and function of pA104R, as well as the immune responses elicited by this antigen. Furthermore, they highlight the potential application of pA104R-specific mAbs and the immunodominant epitope in the study of ASFV-host interactions, as well as the development of vaccines and diagnostic tools.

## MATERIALS AND METHODS

### Cell lines and viral strains

The HEK-293T cells (CRL-3216) and PK-15 cells (CCL-33) were acquired from the American Type Culture Collection. The generous provision of wild boar lung (WSL) cells was made by Professor Guiqing Peng of Huazhong Agricultural University in Wuhan, China. The cells were cultivated under carefully controlled conditions at a temperature of 37°C with a 5% CO_2_ atmosphere. They were nourished in Dulbecco’s Modified Eagle’s medium supplemented with 10% fetal bovine serum (FBS). The primary porcine alveolar macrophage (PAM) cells were prepared from bronchoalveolar lavage according to previously described methods (41) and cultured in RPMI-1640 medium supplemented with 10% FBS. The SP2/0 cells were cultured in RPMI-1640 medium with 10% FBS. The genotype II ASFV isolate strain CN/SD/2019 was propagated in PAM cells.

### Generation and purification of recombinant protein

Whole-length pA104R and a serial of truncated overlapping fragments were amplified (Fig. 4A) and cloned into the prokaryotic expression vectors pET-30a or pGEX-6p-1 following restriction enzyme digestion and ligation. These recombinant plasmids were validated by DNA sequencing and then transformed into *E. coli* BL21(DE3) prior to the induction by isopropyl β-d-1-thiogalactopyranoside at 16°C for 18 h. The His-tag-fused pA104R recombinant protein was expressed and purified by Ni-sepharose agarose. The full-length or truncated pA104R protein expression was confirmed by immunoblotting with His-tag or GST-tag antibodies.

### Animal experiments and ethics statement

Female BALB/c mice (6-week old) were immunized via subcutaneous injection three times with 100 µg purified His-pA104R protein emulsified with an equal amount of Freund’s adjuvant. The final booster immunization was administered via intraperitoneal injection before the following cell fusion procedures. All animal operations followed the regulations of the Administration of Affairs Concerning Experimental Animals in Hubei and the Committee for Protection, Supervision, and Control of Experiments on Animals guidelines of Huazhong Agricultural University (no. HZAUMO-2022-0049). In addition, the mice challenge experiments involving the ASFV strain were conducted in the Animal Biosafety Level 3 laboratory, strictly complying with relevant laws and regulations.

### Preparation of monoclonal antibody

The mAbs were produced as previously described (42). Briefly, spleen cells from mice immunized with His-pA104R three times were fused with SP2/0 myeloma cells using polyethylene glycol (PEG 1450). The fused hybridoma cells were screened in the selective medium supplemented with 20% FBS and hypoxanthine aminopterin thymidine and grown for 10–20 days for the monoclonal cells. Hybridomas that survived were subcloned 3–5 rounds by limiting dilution to obtain stable positive monoclonal cells with the secretion of pA104R-specific antibodies. Positive clones were finally applied to produce ascites containing the mAbs.

### Polyacrylamide gel electrophoresis and immunoblotting

Protein samples were separated by SDS-PAGE and stained with Coomassie Brilliant blue or transferred to polyvinylidene difluoride membranes for the following immunoblotting analysis. The membranes were blocked for 3–5 h with 5% bovine serum albumin (BSA), followed by incubation with primary antibodies overnight at 4°C and then the HRP (Horseradish peroxidase)-conjugated secondary antibodies at 37°C for another 2 h. The chemiluminescence was carried out to visualize the blots.

### IFA (indirect immunofluorescence assay) and IHC (immunohistochemical) analysis

For IFA assays, PK-15 cells transfected with pCAGGS-HA-A104R for 24 h or WSL cells infected with ASFV at 0.1 MOI (multiplicity of infection) for 48 h were fixed with 4% paraformaldehyde and permeabilized with 0.1% Triton X-100. After blocking in 5% BSA, the cells were incubated with each anti-pA104R mAb as the primary antibody and the Alexa Fluor 488-labeled anti-mouse IgG for 1 h at 37°C. After that, the nucleus was finally stained with DAPI and visualized on the EVOS FL Auto system (Thermo Fisher Scientific). For the IHC assay, tissue samples from ASFV-infected pigs fixed in 4% paraformaldehyde were embedded in paraffin. After blocking with BSA, the slides were incubated sequentially with anti-pA104R mAbs, HRP-conjugated anti-mouse IgG, and stained with hematoxylin for visualization.

### Bioinformatics analysis

For conservative analysis, 30 pA104R protein sequences of ASFV isolates were retrieved from the GenBank database and aligned using the ClustalW embedded in GeneDoc software. The spatial characteristics of the immunodominant epitope in pA104R were displayed by mapping the epitope locations on the 3D structure model of ASFV pA104R protein using PyMOL software (37, 43, 44). Protein secondary structure analysis of the pA104R immunodominant epitope was performed using PROTEAN software (DNASTAR Inc.) (44).
